# A Case of Secondary Hemophagocytic Lymphohistiocytosis Presenting with Severe Dyserythropoeisis in a Patient with Autoimmune Hemolysis

**DOI:** 10.1155/2022/8505823

**Published:** 2022-10-19

**Authors:** Kai J. Rogers, Sharathkumar Bhagavathi

**Affiliations:** Department of Pathology, University of Iowa Hospitals and Clinics, Iowa, IA, USA

## Abstract

The diagnosis of hemophagocytic lymphohistiocytosis (HLH) requires that several clinical criteria are met, and often relies on the identification of rare hemophagocytic cells in the bone marrow. Given the challenge in making the diagnosis, additional signs of immune dysregulation in the bone marrow would have practical clinical use in cases where overt hemophagocytosis is not seen. We present here a case of secondary HLH in a patient with autoimmune hemolysis ultimately diagnosed as Evans syndrome that initially presented with profound dyserythropoeisis in both the peripheral blood and bone marrow. We also explore an association between dyserythropoeisis and HLH in a series of cases previously seen at our institution.

## 1. Introduction

Hemophagocytic lymphohistiocytosis (HLH) is a hyperinflammatory syndrome caused by a dysregulated immune response. In all cases, HLH is triggered by an inciting event, often viral illness, and in a subset of cases patients are genetically predisposed to such responses due to deficiencies in cytolytic activity of leukocytes (primary HLH) thereby disrupting the ability to maintain immune homeostasis [1]. Additionally, there exists a subset of secondary HLH called macrophage activation syndrome (MAS or MAS-HLH) which occurs secondary to various autoimmune processes, and which can be clinically indistinguishable from HLH when the inciting event is ambiguous. Given the rarity and nonspecific clinical presentation, HLH is often challenging to diagnose resulting in delayed treatment and increased morbidity and mortality. Here we report a case of secondary HLH that presented with severe dyserythropoeisis in the bone marrow and peripheral blood smear of a patient with Evans syndrome. This case builds on a prior case-series correlating cellular dysplasia with HLH [2] and provides further evidence for this association.

## 2. Case Presentation

**Figure 1:**
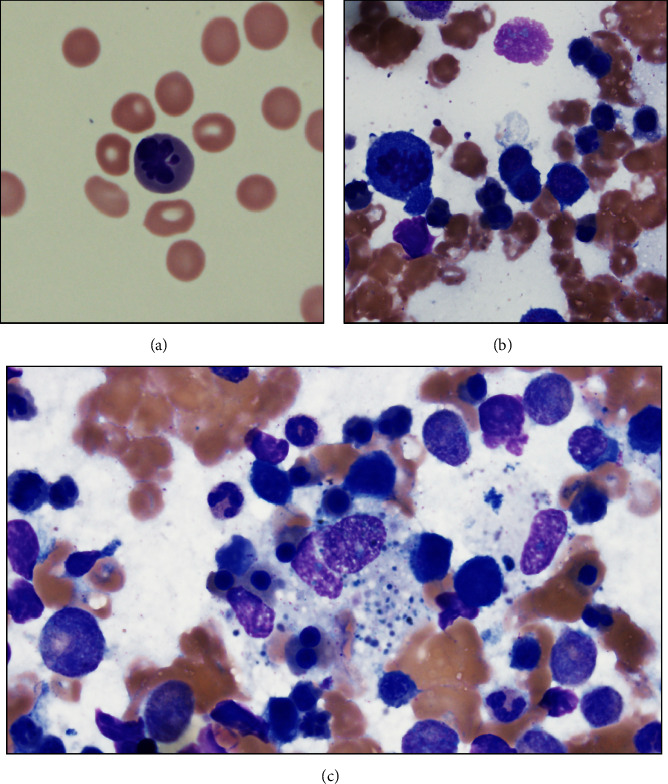
(a) The peripheral blood showed increased nucleated red blood cells with signs of dysplasia (100× objective), (b) the aspirate smears showed profound dyserythropoeisis (50× objective), (c) aspirate smears also revealed readily identifiable hemophagocytic cells, consistent with hernophagocytic lymphohistiocytosis (50× objective).

The patient is a 52 year-old Asian female with a reported history of hepatitis C and nonalcoholic steatohepatitis who presented with 4 days of weakness, back pain, nausea, and vomiting. Upon admission, she was found to be profoundly anemic (Hgb 2.1 g/dl) with a positive direct antiglobulin test (DAT), elevated lactate dehydrogenase, (1,995 U/L) and decreased haptoglobin (<10 mg/dl), initially concerning for acute autoimmune hemolytic anemia. In addition, she had general signs of severe inflammation with ferritin elevated to 75,415 *μ*g/L and elevated c-reactive protein at 15.9 mg/dl, and organ dysfunction with creatinine 1.5 mg/dl, ALT 243 U/L, AST 539 U/L, and total bilirubin 16.5 mg/dl. Shortly after admission, the patient suffered cardiopulmonary arrest, at which point she was stabilized, intubated, and admitted to the medical intensive care unit for further evaluation. After receiving 5 units of packed RBCs, 1 unit of fresh frozen plasma, and 2 units of platelets, a complete blood count was obtained which revealed the following: hemoglobin 7.2 g/dl (normal 11.9–15.5), white blood cell count 11.3 × 10^3^/*μ*l (normal 3.7–10.5), a platelet count of 36 × 10^3^/*μ*l (normal 150–400), and 10 nucleated RBCs/100WBCs (normal 0). Given the overall clinical picture, including multiorgan dysfunction and concern for hemolysis, a bone marrow biopsy was performed. Review of the peripheral blood smear revealed numerous nucleated RBCs, a large subset of which exhibited signs of dysplasia, with abnormal nuclear contours, nuclear budding, and karyorrhexis (Figure 1(a)). The bone marrow aspirates showed a markedly decreased myeloid: erythroid ratio (0.3) and red cell precursors demonstrated profound dysplasia (>50% of cells) including multinucleate forms, nuclear budding, dysmaturation, and karyorrhexis (Figure 1(b)). Granulocyte and megakaryocyte morphology were normal. Given the profound dyserythropoeisis, the initial impression was that this was consistent with a myelodysplastic syndrome, however, on further review of the smears, numerous hemophagocytic histiocytes were identified raising concern for hemophagocytic lymphohistiocytosis (HLH) (Figure 1(c)). As the cytogenetics performed on the bone marrow specimen were normal and no mutations were identified by molecular analysis, MDS was considered less likely, and the bone marrow was ultimately signed out as concerning for HLH based on the presence of hemophagocytic cells. It should be noted that hemophagocytosis has been observed in the setting of recent transfusion and/or hemolysis [3] and thus we recommended this finding be considered in the context of the patient's constellation of symptoms. After review of the biopsy, the patient was found to meet 5/8 criteria for HLH according to the HLH-2004 criteria [4] (Table 1), sufficient for diagnosis, and she improved rapidly on high-dose corticosteroids and intravenous immunoglobulin (IVIG). Given that secondary HLH is a reactive process, a significant workup was performed in the search for an inciting event. From an infectious disease perspective, the patient had no evidence of infection with CMV, EBV, HIV, HAV, HBV, HCV (reported as having a history but PCR negative), or HHV-8. She was, however, found to be PCR positive in a peripheral blood sample for parvovirus B19, and at the time this was presumed to be the underlying cause of her HLH leading to the diagnosis of secondary HLH over MAS-HLH. Of note, the patient was readmitted ∼1 year later with a similar presentation of autoimmune hemolytic anemia and immune-mediated thrombocytopenia. While repeat bone marrow biopsy was not performed, ferritin at the time was elevated to 1,964 *μ*g/L and flow cytometry of the peripheral blood showed increased numbers of CD38+/HLA-DR + CD4+ and CD8+ T-lymphocyte populations which have been shown to be consistent with HLH [5], arguing in favor of the diagnosis, although a definitive workup was not pursued. In addition, given the prior assumption that her HLH was secondary to parvovirus B19 infection, PCR was sent, and she was found to be negative. During her second admission, she was ultimately diagnosed with Evans syndrome, an autoimmune disorder presenting with bicytopenias (commonly autoimmune hemolytic anemia and immune thrombocytopenia) that subsequently triggered the dysregulated immune response manifesting as secondary HLH vs. MAS-HLH. She was treated with high-dose solumedrol and IVIG and later transitioned to eltrombopag with recovery of her RBC and platelet counts. She was discharged and has done well on a combination of eltrombopag and rituximab with no further episodes to date.

## 3. Discussion

As HLH is a clinical syndrome that occurs secondary to dysregulated inflammation, it can be quite difficult to definitively diagnose. Furthermore, as primary HLH is frequently noted during childhood, it is worth noting that the criteria for diagnosis of HLH are often not validated in adult populations which can lead to misdiagnosis in patients with ambiguous presentations [6]. Patients who raise clinical concern for HLH are often critically ill and fall just short of meeting the diagnostic criteria, leading the clinical team to order a bone marrow biopsy and request pathologists to evaluate for the presence of hemophagocytic cells. Such cells are often challenging to find and clear evidence of hemophagocytosis is uncommon, leading to inconclusive results that can delay treatment while awaiting the results of send-out tests with a slow turnaround time of several days such as NK cell activity and soluble IL-2 receptor levels. As such, additional signs and symptoms that consistently predict HLH are of high clinical interest. In this endeavor, a 2000 study by Imashuku et al. was the first to suggest a connection between HLH and reactive dysplasia [7], followed shortly by a 2001 case-series focusing specifically on dyserythropoeisis [2]. This correlation was subsequently observed in the context of Kikuchi disease [8], and we report our experience here in order to strengthen this connection.

To briefly evaluate the possible association between dyserythropoeisis and HLH, we retrospectively reviewed prior cases of HLH at our institution and found dyserythropoeisis in 9/22 cases of secondary HLH that received a bone marrow biopsy (40.1%). In contrast to the case we present here, the dyserythropoeisis was typically less severe, and in no case were dysplastic cells observed in the peripheral blood, a striking and unique feature of our case. Interestingly, in 5/9 cases meeting criteria for HLH with dyserythropoeisis, no hemophagocytic cells were identified, leaving dysplasia as the only morphologic evidence of disease. This is an important point as obvious dysplasia is easily identified and may serve as a marker of profound bone marrow stress providing evidence supporting the diagnosis and prompting a more thorough review when obvious hemophagocytic cells are not initially identified. It is also worth noting that other groups have identified myeloid neoplasms which may also present with dyserythropoeisis such as MDS, AML, and MPNs as triggers for HLH [9, 10] making their exclusion an important part of the diagnostic workup.

## 4. Conclusion

This case describes a rarely reported association between dyserythropoeisis and secondary HLH that has been observed in ∼40% of cases at our institution. Given the small number of reports to date, the mechanistic link between HLH and cellular dysplasia remains unexplored, although future work in this area may prove insightful. While evidence of hemophagocytic cells remains the key diagnostic feature of HLH from the pathologist's perspective, it is important to note that any abnormality not directly attributable to another disease process should prompt consideration of HLH in the appropriate clinical context.

## Figures and Tables

**Table 1:** Diagnostic criteria for HLH in our patient.

Diagnostic criteria	Patient value	Criterion met?
Fever ≥38.5°C for >7 days	Patient was afebrile, Tmax = 37.4°C	No
Splenomegaly	12.3 cm craniocaudal length by ultrasound	No
Cytopenia of 2+ cell lines	Bicytopenia (Hgb nadir of 5.8 g/dL, platelet nadir of 18,000 cells/*μ*L)	Yes
Hypertriglyceridemia (fasting triglycerides >265 mg/dL)	437 mg/dL	Yes
Hemophagocytosis in bone marrow, spleen, lymph node, or liver	Extensive hemophagocytosis in bone marrow	Yes
Low or absent NK activity	Non-detectable	Yes
Serum ferritin concentration ≥500 *μ*g/L	Patient value ranged from 2,759 to 75,415 *μ*g/L	Yes
Soluble CD25 (IL-2 receptor) ≥2400 U/mL	1,035 U/mL, reference range 137–838 U/mL	No

## Data Availability

No data were generated for this case report. Any additional information regarding the case is available from the corresponding author upon request.
